# Titania-mediated stabilization of fluorescent dye encapsulation in mesoporous silica nanoparticles†

**DOI:** 10.1039/d4na00242c

**Published:** 2024-05-29

**Authors:** Laura Spitzmüller, Jonathan Berson, Fabian Nitschke, Thomas Kohl, Thomas Schimmel

**Affiliations:** a Geothermal Energy and Reservoir Technology, Institute of Applied Geosciences, Karlsruhe Institute of Technology Karlsruhe Germany laura.spitzmueller@kit.edu; b Institute of Nanotechnology, Karlsruhe Institute of Technology Hermann-von-Helmholtz-Platz 1 76344 Eggenstein-Leopoldshafen Germany; c Institute of Applied Physics, Karlsruhe Institute of Technology Wolfgang-Gaede-Straße 1 76131 Karlsruhe Germany

## Abstract

Mesoporous silica nanoparticles hosting guest molecules are a versatile tool with applications in various fields such as life and environmental sciences. Current commonly applied pore blocking strategies are not universally applicable and are often not robust enough to withstand harsh ambient conditions (*e.g.* geothermal). In this work, a titania layer is utilized as a robust pore blocker, with a test-case where it is used for the encapsulation of fluorescent dyes. The layer is formed by a hydrolysis process of a titania precursor in an adapted microemulsion system and demonstrates effective protection of both the dye payload and the silica core from disintegration under otherwise damaging external conditions. The produced dye-MSN@TiO_2_ particles are characterized by means of electron microscopy, elemental mapping, ζ-potential, X-ray diffraction (XRD), nitrogen adsorption, Thermogravimetric analysis (TGA), fluorescence and absorbance spectroscopy and Fourier Transform Infrared Spectroscopy – Total Attenuated Reflectance (FT-IR ATR). Finally, the performance of the titania-encapsulated MSNs is demonstrated in long-term aqueous stability and in flow-through experiments, where owing to improved dispersion encapsulated dye results in improved flow properties compared to free dye properties. This behavior exemplifies the potential advantage of carrier-borne marker molecules over free dye molecules in applications where accessibility or targeting are a factor, thus this encapsulation method increases the variety of fields of application.

## Abstract 2

A robust and stable encapsulation method for mesoporous silica nanoparticles, protecting the payload, preventing leakage and stabilizing the nanoparticles.

## Graphical abstract

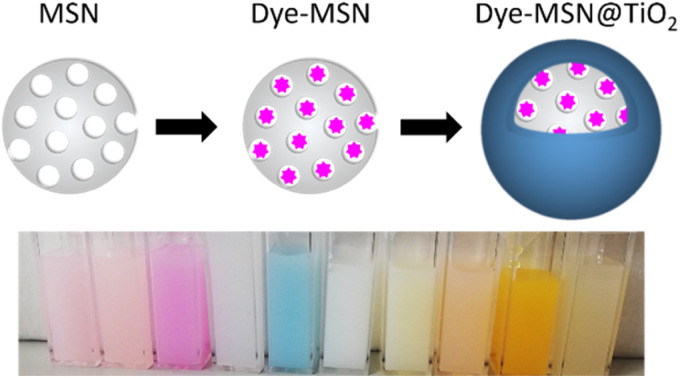

## Introduction

Mesoporous silica nanoparticles (MSNs) emerged as versatile carriers for a wide variety of guest molecules, significantly advancing many research fields from nanomedicine, biotechnology, pharmaceutics and catalysis to environmental sciences including reservoir technology for renewable energies.^1–4^ Their well-defined pores with tunable sizes, large surface areas, and ease of functionalization make MSNs ideal for a variety of applications.^5^ MSNs function as high-capacity carriers, useful for loading molecules and transporting them to specific locations or through preferred paths. Their intricate porous structure allows for the concentration and entrapment of a variety of molecules, ranging from therapeutic agents to diagnostic markers.^6^ The MSNs can be designed to carry their payload throughout their application or to release it at an opportune moment or location in response to a specific and predetermined external stimulus. Carrier MSNs can be directed to a desired location within biological or geological systems or across various mediums, leveraging the targeting capabilities conferred by surface modifications.^7–9^ Such targeted transport is crucial in applications where the mere presence of concentrated molecules at a specific site can induce the desired effect, such as in imaging or sensing.^10^ In contrast, release MSNs are based on the ability to release the payloads at targeted locations, which is particularly advantageous for targeted drug delivery. This capability hinges on stimuli-responsive release mechanisms where the MSNs are engineered to respond to specific environmental triggers—such as pH changes, temperature fluctuations, or enzymatic activities prevalent at the target site.^5,11–13^ The controlled release ensures that the therapeutic agents exert their action precisely where needed, minimizing systemic distribution and associated side effects. This precision in payload release from MSNs underscores a significant advancement in nanomedicine, offering a more targeted and effective therapeutic strategy.^14^

Whether the MSN system is designed to release its payload or retain it throughout the process, efficient encapsulation is essential. It has to prevent leakage of the loaded molecules, and protect them from premature degradation due to prevalent conditions outside of the host nanoparticle but also ensures their controlled release at the target site.^15^ The process of encapsulation itself may be challenging, as the chemical modifications required to form the encapsulating layer may harm the stability and functionality of the payload.

The inherent instability of MSNs in aqueous media is a double-edged sword in the realm of nanotechnology applications. In aqueous environments, unmodified silica nanoparticles tend to dissolve within hours.^16,17^ The silica dissolution and its rate depend on ambient factors such as temperature, pH-value and ionic strength of the solution, the presence of specific ions and chemicals as well as on properties of the silica itself such as degree of condensation, size and porosity.^15–18^ This instability can be advantageous in fields like biomedicine, where controlled dissolution can be harnessed for timed release of therapeutic agents or creating clearance pathways for excretion of the treatment and its metabolites.^10,19,20^ Conversely, it poses a challenge in applications requiring long-term structural integrity, such as in subsurface reservoir tracing (*e.g.* for hydrology, for oil and gas industry and as geothermal tracer), structural composites or as catalyst supports where premature dissolution would compromise functionality.

A strategic application of an encapsulation layer can slow the dissolution by restricting access of water molecules to the silica surface, thereby modulating the release kinetics of encapsulated substances.^21^ A non-porous layer can provide an even more significant barrier, potentially halting the dissolution altogether.^22^ Such encapsulation not only extends the range of environments in which silica nanoparticles can be utilized but also has the potential to fine-tune their dissolution kinetics for specific applications, enhancing their versatility and performance across various scientific and industrial fields.^16^

In this work, we present an approach that utilizes a titania (TiO_2_) layer to encapsulate molecules within MSNs. Titania is robust and stable and exhibits low solubility in water under a wide range of pH values, properties which make it ideal for applications where (aqueous) long-term stability and/or endurance under harsh ambient conditions is needed (*e.g.* imaging, environmental tracing).^23,24^ By forming a protective titania layer over the encapsulated molecules, it is possible to enhance the stability and prevent leakage of the payload.

Titania can be applied to coat silica nanoparticles by either chemical or physical deposition techniques. Many different synthetic routes exist, using hydrothermal and/or microemulsion synthesis with various titania precursors,^25–28^ in either alkaline or acidic environments or as delayed co-condensation reaction with silica forming a silica-titania mixed layer. Other deposition methods include vapor deposition^29^ of a titania layer or nanoparticles or producing aerosols.^30^ The synthesis parameters greatly affect the subsequent thickness, porosity, uniformity and crystalline structure of the formed layer, which in turn has a significant influence on the performance of the titania in terms of light transparency and scattering and stability. For the functionality of the particles in imaging and as fluorescent hydrological or geothermal tracers it is however pivotal to minimize the suppression of the optical signal of the encapsulated molecules, *i.e.* the titania needs to be sufficiently transparent in the range of visible light. Additionally, the titania coating is supposed to act as pore blocking agent and stabilization of the silica carrier against dissolution effects.

Moreover, for long-term applications (for example “geoimaging”, tracer technology in groundwater and reservoir exploration) it is crucial having a carrier that is not prone to degradation and additionally having ideally a pore blocking agent that prevents leaching of the loaded dye while also ensuring the integrity of the carrier. While titania has already been utilized as a thin film or coating layer for *e.g.* increasing biocompatibility by altering the surface of drug loaded MSN,^29^ the robust and stable nature of titania has not been exploited for payload encapsulation applications.

The presented synthesis is applicable to encapsulate a wide variety of guest fluorescent dyes and the titania acts as an effective pore blocking agent for the tested mesoporous silica nanoparticles carriers. The dye-MSN@TiO_2_ particles are characterized by electron microscopy, fluorescence and absorbance spectroscopy, infrared spectroscopy, X-ray spectroscopy and ζ-potentiometry. Successful encapsulation in proved by long-term stability experiments and flow-through experiments. Additionally, effect of titania-coating is compared to commonly applied hydrophobic and hydrophilic pore blockers.

## Experimental

### Synthesis

#### Materials

The following chemicals were used without further purification: cetrimonium bromide (CTAB, Alfa Aesar 98%), tetraethyl orthosilicate (TEOS, Sigma Aldrich 99%), tetrabutyl orthotitanate (TBOT, Sigma Aldrich, synthesis grade), (3-mercaptopropyl)trimethoxysilane (MPTS, BLD pharm 95%), PEG-maleimide (BLD pharm 97% av. purity), *n*-octadecyltrimethoxysilane (C18, ABCR GmbH, 95%), dotriacontane (“paraffin” Sigma Aldrich, 97%), sodium dodecyl sulfate (Sigma Aldrich ≥99%), potassium chloride (KCl, VWR GPR Rectapur >99%), sodium chloride (NaCl VWR GPR Rectapur ≥99%) phosphate-buffered saline (PBS, VWR life science, biotechnology grade, pH 7.4), Millipore water (>18 MΩ), ammonia (NH_4_OH, Merck, 28–30%), ethanol (VWR Chemicals, AnalaR Normapur ≥99.8%), acetone (VWR Chemicals, AnalaR Normapur, ≥99.8%) dry acetonitrile (Merck, 99.5%), cyclohexane (VWR Chemicals, AnalaR Normapur ≥99.5%), *n*-hexanol (VWR GPR Rectapur ≥98%), Triton X-100 (Sigma Aldrich for analysis), sulfuric acid (H_2_SO_4_, Merck Supelco 98%), absolute toluene (VWR Chemicals, AnalaR Normapur ≥99.5%), hexane (Carl Roth, 99%), hydrochloric acid (HCl, Honeywell Fluka 36.5–38%), sodium hydroxide (NaOH, Merck Emsure ≥99%), nitrogen (<10 ppm O_2_). The following dyes were used without further purification: coumarin 307 (C307, Radiant Dyes Chemie), sodium fluorescein (fluo, Sigma Aldrich), rhodamine 6G (R6G, Sigma Aldrich for fluorescence), rhodamine B (RhB, Sigma Aldrich for fluorescence), sulforhodamine G (SG, Sigma Aldrich 60% dye content), tris(2,2-bipyridyl)dichlororuthenium(ii) hexahydrate (Ru(bpy)_3_^2+^, Acros organics, 98%,) and rhodamine 800 (R800, Sigma Aldrich).

#### Synthesis of carrier

Mesoporous carrier synthesized according to Huang, *et al.*^31^ Briefly, 109 mg cetrimonium bromide (CTAB) is mixed with 54 mL Millipore water and 1.194 mL ammonia (NH_4_OH) added. After stirring for 1 hour, 0.465 mL tetraethyl orthosilicate (TEOS) is added dropwise, followed by 5 hours at high stirring rate. Finally, the particles undergo several wash-centrifugation cycles with water and ethanol and are dried in vacuum. The template (CTAB) is removed *via* calcination at 550 °C at a heating rate of 1 °C min^−1^ over 6 hours.

#### Encapsulation of dyes

Encapsulation of dyes was performed in slightly altered version of Rudolph, *et al.*^9^ 4 mg dye per 10 mg particles were added to the calcined particles, and stored overnight in a glovebox under nitrogen atmosphere. Then, 0.5 mL dry acetonitrile per 10 mg particles is added and the particle dispersion is stirred overnight in glovebox.

### Surface modifications

#### Coating with titania

The particles are taken out of glovebox and retrieved by centrifugation. After washing with hexane, the particles are dried in vacuum, weighted (usually 50 mg) and redispersed in 4.8 mL Millipore water using a sonotrode. Titania coating was performed using a reverse water-in-oil microemulsion method adapted from Fu and Qutubuddin.^32^ Briefly, 15 mL cyclohexane was mixed with 3.6 mL 1-hexanol and 3.44 mL TritonX-100. After stirring for 1 minute, the particle dispersion is added followed by dropwise addition of 307 μL TBOT. After further stirring for 20 minutes, 60.9 μL H_2_SO_4_ (98%) is added to the microemulsion and stirred for 48 hours. The microemulsion is broken by addition of excess amount of acetone, followed by washing-centrifugation cycles with acetone, ethanol, ethanol–water, and water.

#### Pore blocker

Two different type of pore blockers were chosen; a hydrophilic polymer (poly(ethylene) glycol, PEG), and a hydrophobic paraffin.

##### PEG

The particles are removed from the glovebox and retrieved by centrifugation. After washing with hexane, the particles are dried in vacuum. PEGylation was performed adapting von Baeckmann, *et al.*^33^ Briefly, the dried particles are mixed with absolute toluene and heated under inert atmosphere to 100 °C while stirring moderately. After 3 hours, thiol-functionalization was performed by adding 12 mM (3-mercaptopropyl)trimethoxysilane (MPTS) per gram particles and stirred further for 4 hours under inert atmosphere at 100 °C. After, the particles are retrieved by centrifugation and dried in vacuum. For PEGylation, PEG-maleimide was used. The thiol-maleimide click chemistry reaction was performed as described by von Baeckmann, *et al.*^33^ Briefly, 75 mg of thiol-functionalized nanoparticles are suspended in 5 mL Millipore water and stirred vigorously overnight. After, 0.5 mL phosphate buffered saline (PBS) is added to the solution, followed by 75 mg PEG-maleimide. The solution is stirred overnight at ambient temperature. Finally, the particles are retrieved by centrifugation and washed at least three times with water until supernatant remains clear. After drying in vacuum, the particles are ready to use.

##### Paraffin

After dye encapsulation, particles dispersed in acetonitrile are removed from the nitrogen atmosphere in the glovebox and 75 μL *n*-octadecyltrimethoxysilane (C18) per 10 mg particles is added. The solution is stirred overnight under ambient conditions. The particles undergo several washing-centrifugation cycles with acetonitrile and hexane and are eventually dried. For paraffin coating, dried particles are redispersed in 8 mL hexane and 75 mg dotriacontante is added per 10 mg particles. The solution is sonicated for 15 minutes, followed by stirring for 15 minutes. The particles are retrieved by centrifugation followed by washing-centrifugation cycles with a mixture of SDS-water and water until supernatant is clear.

### Analytical devices

Samples were characterized using SEM, TEM, nitrogen absorption (BET surface measurement), TGA, FT-IR ATR, XRD, ζ-potentiometry, fluorescence and absorbance spectroscopy.

#### Scanning electron microscopy (SEM)

A Zeiss Leo 1530 was used to analyze size, shape and size distribution of the nanoparticles. Operated in InLens and SE2 mode.

#### (Scanning) transmission electron microscopy ((S)TEM) and elemental mapping

(S)TEM and elemental mapping was performed using a ThermoFischer Themis 300 probe-corrected (S)TEM with 300 kV.

#### Brunauer–Emmett–Teller (BET) surface measurement

For BET measurements, the samples were degassed at 90 °C overnight in vacuum and surface area was determined by nitrogen adsorption with a Quantachrome Nova 4000e.

#### Thermogravimetric analysis (TGA)

TGA measurements were performed with a STA 409 PC Netzsch under atmospheric conditions with a heating rate of 10 °C min^−1^ up to 600 °C. The results were normalized to the relevant weight loss percentage expected for the respective dye.^34^

#### Fourier transform infrared spectroscopy – attenuated total reflectance (FT-IR ATR)

FT-IR ATR analysis was performed using a Nicolet iS50 with wavenumbers from 4000 to 400 cm^−1^. Measurements were repeated twenty times. Samples were measured in dry conditions.

#### X-ray diffraction (XRD)

The powder X-ray (XRD) data of the samples were collected using a PANalytical X'Pert Pro X-ray diffractometer with Cu Kα radiation. The angle range between 5 and 80° (2*θ*) was recorded over 2 hours at room temperature with a step size of 0.01° s^−1^.

#### ζ Potentiometry

ζ-Potentials were retrieved measuring electrophoretic potential with a Malvern Zetasizer NanoZS using a folded capillary cell. Analyses were performed in 10 mM KCl solutions at pH 1.8, 4.4, 6.6, 8.5 and 9.5 m at 25 °C. pH-values were adapted by addition of HCl or NaOH. Measured electrophoretic potential of the particles was automatically converted to ζ-potential values using Smoluchowski approximation. Generally, ζ-potential represents net electrical charge in the shear plane and are result of surface charge of particles, attached oppositely charged ions in the Stern layer and diffuse double layer. Therefore, ζ-potential can serve as approximation to surface charge of particles. However, they are strongly dependent on temperature, ionic strength, and pH.

#### Fluorescence and absorbance spectroscopy

Fluorescence was measured using a Cary Eclipse spectrofluorometer, UV-Vis absorbance was measured with an UV-VIS PerkinElmer Lambda 9 spectrometer. For both analyses, cuvettes with an optical path length of 1 cm were used. Photodegradation of fluorescence was tested by exposing the samples in sealed quartz cuvettes to UV-light (lamp specification 400W, MHL570). Reduction in fluorescence due to lower quantum yield of loaded dye molecules and adsorption and scattering of the particle matrix was determined by fluorescence spectroscopy comparing free dye and encapsulated molecules. The concentration of the dye solution used for this comparison was determined according to the calculated amount of loaded dye per particle, based on the results of the TGA analysis.

#### Flow-through experiments

A 25 cm glass column, with 0.8 cm inner diameter was filled with washed and dried (105 °C) coarse quartz sand (size 1.0–1.6 mm) and saturated with deionized water using a peristaltic pump. Flow rate was measured 5x over 30 s to be 40 mL min^−1^. The glass column was connected to a 3.3 × 1.0 × 1.0 cm^3^ quartz flow-through cuvette. Fluorescence was measured automatically with a frequency of 1 Hz giving a continuously measured breakthrough curve of dye, dye-MSN and dye-MSN@TiO_2_. Dye and particle concentration were measured in advance to obtain a calibration line to convert fluorescence intensity into concentration. With the concentration and flow-through velocity, retrieval rates and *vice versa* retention of dye and particles can be calculated.

## Results and discussion

### Synthesis of dye-doped mesoporous silica nanoparticles

**Fig. 1:**
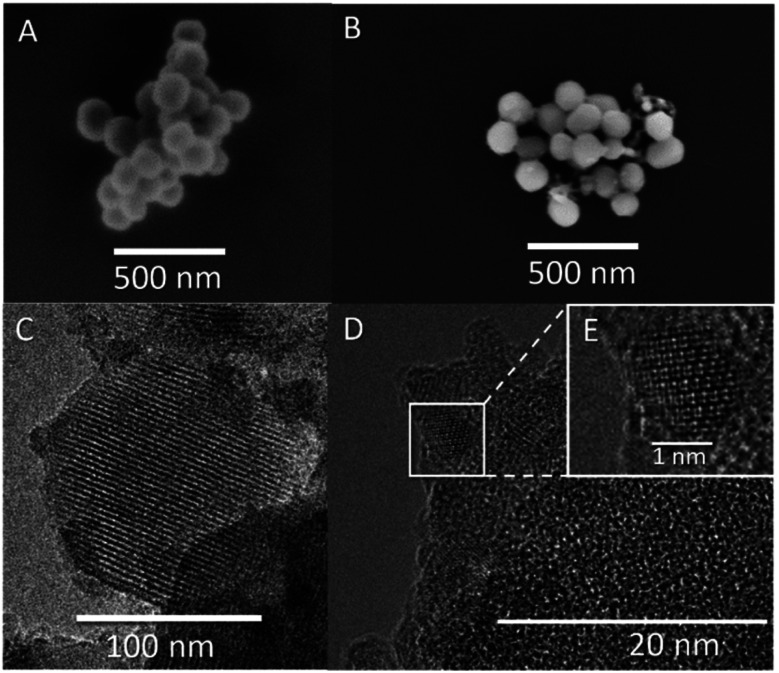
SEM and TEM images of MSN and dye-MSN@TiO_2_ particles. (A) and (B): SEM image of pristine MSN (A) and dye-MSN@TiO_2_ (B). (C) Shows pore structure of MSN, (D) shows interface of MSN and titania layer. Inset picture (E) shows crystal lattice of titania in the shell. Titania crystallites show sizes of 3–4 nm.

Mesoporous silica nanoparticle carrier was produced using modified Stöber synthesis with CTAB-micelles as the sacrificial organic template.^31^ Resulting carrier size was determined to be 142 ± 24 nm (Fig. 1A and C), with mean pore sizes of 2.7 nm (Fig. 1C), a BET surface area of 868 m^2^ g^−1^ and ζ-potential in 10 mM KCl at pH 6.6 of −18.8 mV ± 4.2 mV. To ensure long-term hydrothermal stability, the carrier was calcined. Spitzmüller, *et al.*^16^ showed that calcination is able to re-order silanol bonds to siloxane bonds on the silica surface, thus increasing hydrothermal stability. Fluorophore-doping was adapted from Rudolph, *et al.*^9^ and resulting dye concentration inside the MSN carrier was determined to be approx. 19 mg g^−1^ using TGA, comparable to previously reported MSN loading capacities of 5–70 mg g^−1^.^35,36^ In this study, we deliberately used fluorophores that were contained in the pores but were not covalently bound to the silica matrix, both in order to show the generality of the encapsulation method and to be able to demonstrate adequate pore blocking functionality. Since fluorophore molecules were not chemically bond to the MSN matrix, sonication or dissolution in water would remove unbound fluorophore by diffusion unless this is prevented by a stable and leakage proof coating.

### Titania coating of mesoporous carrier

Titania coating was performed adapting a reversed microemulsion synthesis (water-in-oil, W/O) from Fu and Qutubuddin.^32^ Firstly, dye-impregnated mesoporous silica nanoparticles were well dispersed in water using a sonotrode. The amount of required water was derived using the ratio between water-surfactant and microemulsion droplet size.^37,38^ Even though the silica condensation reaction was performed with alkaline catalysis, acidic conditions were chosen for the growth of the titania layer, as this was found to be the most controlled and selective process, yielding highly reproducible results. Concentrated sulfuric acid was used to acidify the water phase of the emulsion due to its low water content, so that addition of the acid would have negligible influence on the size of the water-in-oil droplets. The titania precursor that was chosen was tetrabutyl orthotitanate (TBOT), which likewise ensure high reproducibility of the hydrolysis products. The usage of a strong acid gives two major implications. First, the silica nanoparticles are prevented from degradation due to acidic environment,^16^ which implies that the network remains intact during synthesis process. Second, some fluorophores are sensitive to acidic pH such as sodium fluorescein. We therefore advise to protect these fluorophores by growing a silica shell using the microemulsion method as described in Rudolph, *et al.*^9^ prior of growing a titania shell in order to prevent fluorescence loss. By addition of a protective silica shell, the fluorophore inside the pores is shielded against the acid and maintains its fluorescence. However, solely using a silica shell does not sufficiently stabilize the particles against disintegration and still a protective titania layer is demanded. As example, we performed a fluo-MSN@SiO_2_@TiO_2_ synthesis (ESI Fig. S1†) yielding stable, protected fluorescent particles. Other dyes such as rhodamines do not require this additional step,^39^ and we therefore focused on using dyes which are not sensitive to acidic environments. Resulting dye-MSN@TiO_2_ particles show sizes of 151 ± 38 nm (determined by SEM, Fig. 1B and D). The BET surface area is reduced from 868 m^2^ g^−1^ to 469 m^2^ g^−1^, both values being in agreement with open and capped MSN, respectively.^29,40,41^

### (S)TEM and elemental mapping/electron microscopy

**Fig. 2:**
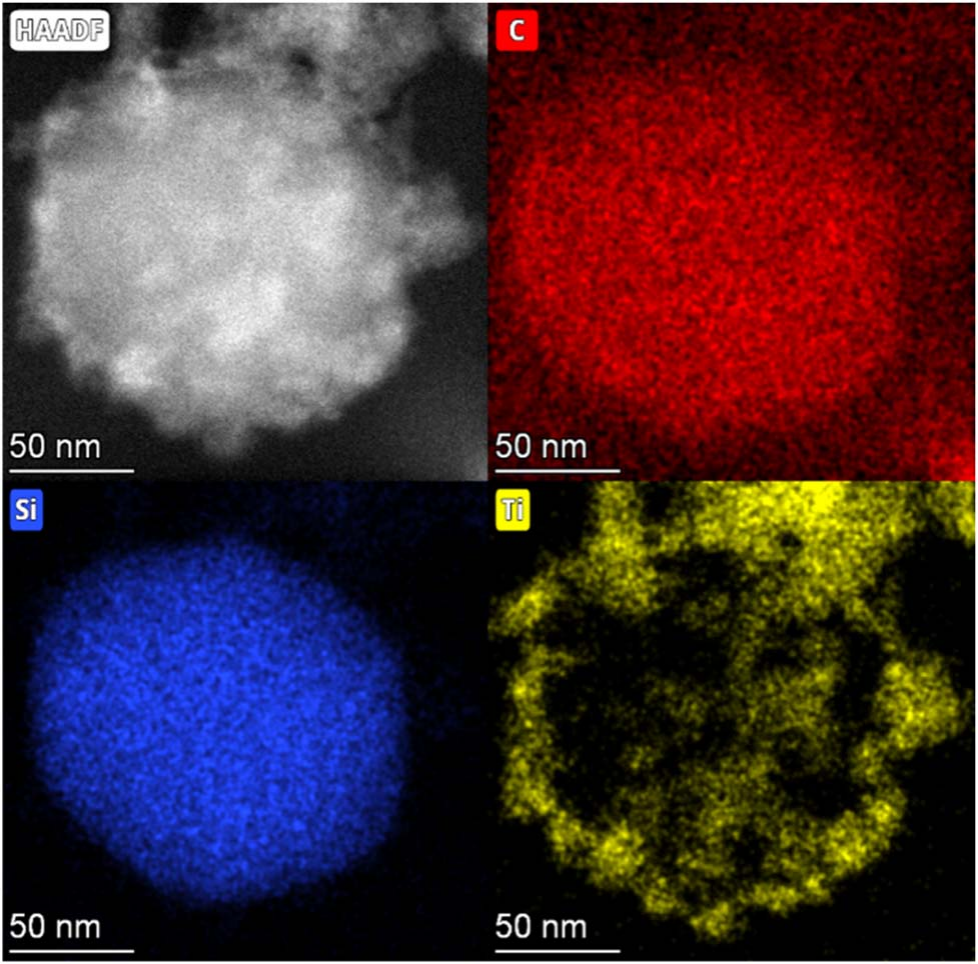
STEM image of dye-MSN@TiO_2_ (top left) and the corresponding elemental mapping. Carbon (top right, carbon signal emanates from the R800 dye inside the particle with a slight background signal from the carbon tape), silicon (bottom left) and titanium (bottom right).

STEM analysis of the MSN revealed ordered hexagonal pore shape with sizes of 2–3 nm (Fig. 1C) which is in agreement with previous observations.^31^ The titania layer thickness is measured to be 7.1 nm ± 4.3 nm (*n* = 36) and shows a mixture of amorphous titania and anatase nanocrystallites with 3–4 nm average size (Fig. 1D). Fig. 1E shows a zoom on a titania crystallite in the titania layer with a clearly discernible crystal lattice. Elemental mapping further confirmed the presence of a silica core and a titania shell (Fig. 2) with atomic fractions of 67.91 ± 3.6% oxygen, 27.31 ± 3.82% silicon and 4.78 ± 0.81% titanium. The presence of dye inside the MSN is indicated by carbon accumulation (Fig. 2, corresponding to the presence of the R800 dye, C_26_H_26_ClN_3_O_5_ inside the pores).

### ζ-Potential

**Fig. 3:**
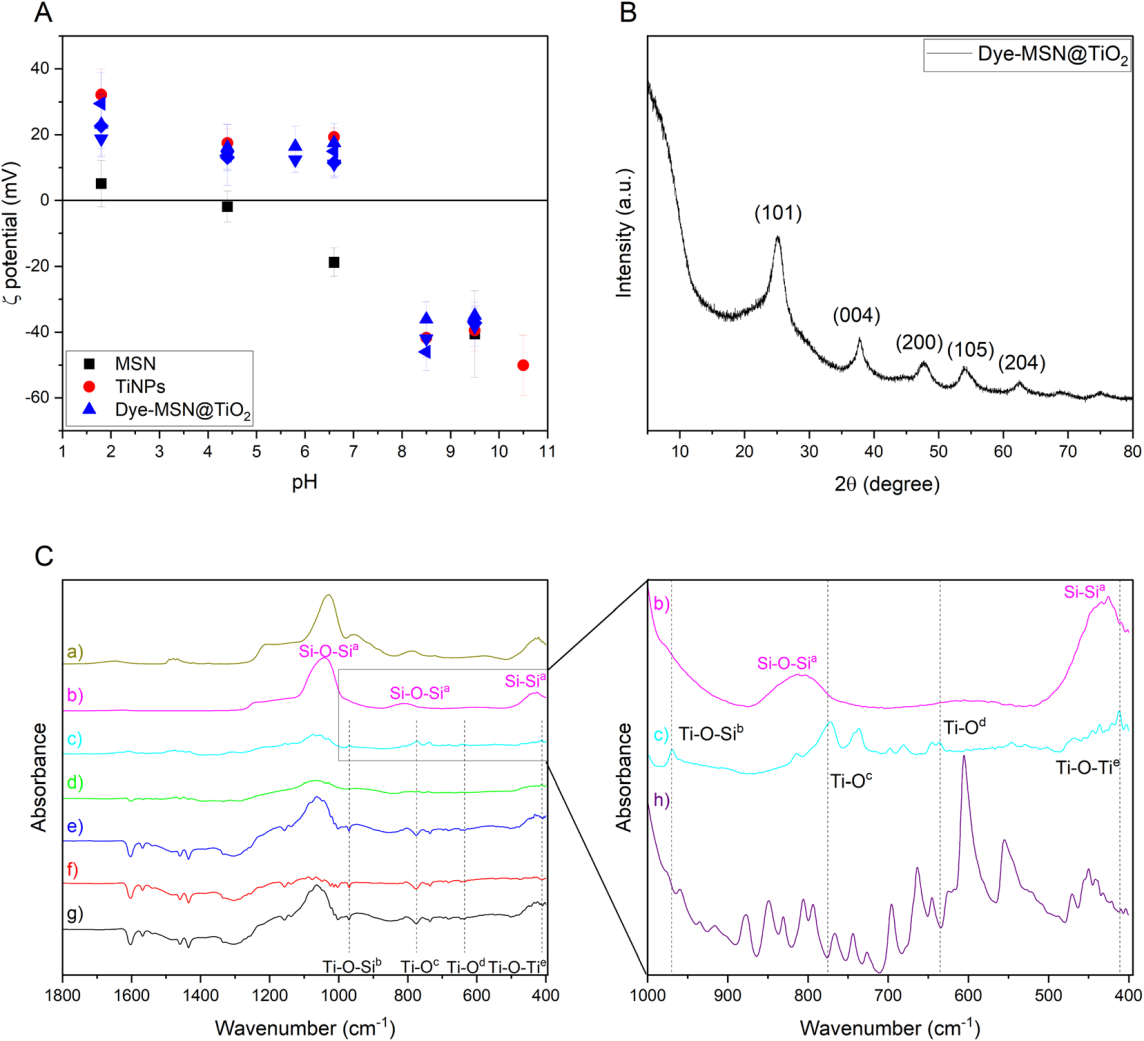
Overview of characterization methods. (A) ζ-Potential evolution over pH for MSN (black squares), titania nanoparticles (red dots) and various dye-MSN@TiO_2_ (blue symbols). (B) Representative XRD pattern of Dye-MSN@TiO_2_. Peaks can be assigned to anatase, a polymorph of titania. (C) ATR absorbance spectra of CTAB-MSN (a), calcined MSN (b), SG-MSN@TiO_2_ (c), fluo-MSN@SiO_2_@TiO_2_ (d), R800-MSN@TiO_2_ (e), RhB-MSN@TiO_2_ (f), R6G-MSN@TiO_2_ (g). Zoom-in on the 1000–400 cm^−1^ region marked with a grey box in (C). Comparison of pristine, calcined MSN (b) to SG-MSN@TiO_2_ (c) and spectra of SG-dye (h). Literature sources for peak assignment ^a^ Socrates,^64^^b^ Zu, *et al.*,^65^^c^ Islam, *et al.*,^61^^d^ Hema, *et al.*,^63^^e^ Pérez, *et al.*^66^

ζ-Potential evolution over pH-range for pristine MSN (black square), pure titania (red dot) nanoparticles and various dye-MSN@TiO_2_ (blue symbols) is displayed in Fig. 3A. Pristine MSN carrier and pure titania particles show an isoelectric point (IEP) between 1.8 and 4.4 and 6.6 and 8.5 respectively, which is in agreement with data from similar nanoparticle systems.^30,42–44^ The dye-MSN@TiO_2_ nanoparticles consist of both silica and titania. Consequently, one could expect the ζ-potential to be in range between these two “end-points”.^45,46^ In fact, ζ-potential variations are decent indicators in assessing titania-coating efficiency on silica nanoparticles.^43^ Wilhelm and Stephan^43^ observed a slight shift of IEP from 6.7 for pure titania to 5.7–6.7 for titania-coated silica nanoparticles and only minor differences in absolute ζ-potential. For our dye-MSN@TiO_2_, ζ-potential evolution and IEP also resembles titania more than pristine MSN. Hence, two major implications can be drawn: the coating of MSN with titania was successful and the coating layer further effectively shields the core (MSN and dye) as there is also no significant difference between the measured potential of different incorporated dyes, regardless of their charge (anionic, cationic).

### XRD

XRD was used to analyze the titania properties. A representative XRD pattern is shown in Fig. 3B. The broadness of the peaks is either a sign of amorphous nature of the samples or of small crystallite size, which can be expected from sol–gel process without subsequent heat treatment.^28,47–49^ The peaks at 25.1°, 37.8°, 47.8°, 54°, 62.6° can be assigned to the (101), (004), (200), (105), and (204) planes of anatase (TiO_2_ polymorph), respectively.^50^ Amorphous silica diffraction peak is in the 2*θ* range between 23° and 25°,^51^ possibly leading to the broadening of the anatase diffraction peak at 25.1°. Crystallite size of anatase particles can be estimated to be 3–4 nm using Scherrer equation which is confirmed by TEM data (Fig. 1E). Anatase has a band gap of 3.2 eV, *i.e.* is transparent for visible light.^52,53^ As result, titania is applicable as coating layer for the fluorophore-doped mesoporous silica nanocarriers as long as the excitation–emission wavelengths of the loaded fluorophores lay outside of the UV-range.

### FT-IR ATR

Fig. 3C shows FT-IR ATR absorbance spectra of MSN and various dye-MSN@TiO_2_. Successful template removal through calcination is proven (CTAB-MSN (a) to calcined MSN (b)). Additionally, the disappearance of Si–OH bonds at 950 cm^−1^ indicate higher hydrothermal stability of the synthesized silica nanoparticles due to a higher degree of condensation.^16^Fig. 3C(c)–(g) show ATR spectra of SG-MSN@TiO_2_, fluo-MSN@SiO_2_@TiO_2_, R800-MSN@TiO_2_, RhB-MSN@TiO_2_ and R6G-MSN@TiO_2_. The spectra reveal the characteristic Si–O stretching, bending and rocking vibrations at 1100 cm^−1^–1000 cm^−1^, 811 cm^−1^ and 430 cm^−1^, respectively.^54^ Any additional peak originate either from the dye or to the titania. As shown in zoom-in on Fig. 3C, absorbance spectra of dye-MSN@TiO_2_ are mostly a superimposition of the silica carrier (a) and the spectra of the dye (h). Consequently, non-assigned peaks must originate either from titania coating process chemicals, from titania bonds or from interaction of titania and silica. Generally, the spectra intensity is lowered which can be attributed to the presence of titania.^55^ Studies show Si–O–Ti bonds in the region of 960–910 cm^−1^, Ti–O stretching and Ti–O–Ti bending vibrations at around 770 cm^−1^, sym. Ti–O–Ti stretching at 411 cm^−1^ and deformation vibration of Ti–O–Ti in the area 1200–1000 cm^−1^.^56–61^ The peak observed at 960 cm^−1^ in all dye-MSN@TiO_2_ might therefore indicate Si–O–Ti but could be also be assigned to Si–OH bonds. However, since there is no peak at the MSN carrier (Fig. 3C) the appearance of the bond might indicate Si–O–Ti bonds rather than Si–OH bonds. This assumption can be based on data from Zhuravlev^62^ that showed slow rehydroxylation (*i.e.* Si–OH bonds) of calcined silica surfaces (*i.e.* Si–O–Si bonds) under ambient conditions. Furthermore, the peaks at 770 cm^−1^, 635 cm^−1^ and 411 cm^−1^ can be ascribed to Ti–O stretching and Ti–O–Ti bending, Ti–O vibrations and sym. Ti–O–Ti stretching.^57–59,63^ In summary, the ATR spectra of the dye-MSN@TiO_2_ particles show silica-related, dye-related, titania-related vibrations as well as additionally silica-titania bonds, supporting the observation that dye-encapsulation by titania coating was successful.

### Fluorescence and absorbance spectroscopy and UV-sensitivity

**Fig. 4:**
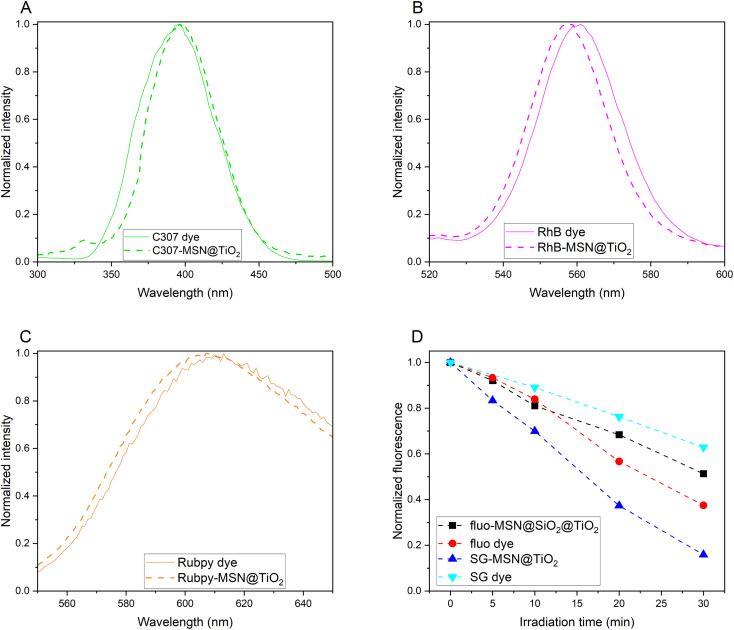
Normalized fluorescence intensity of coumarin 307 and C307-MSN@TiO_2_ (A), rhodamine B and RhB-MSN@TiO_2_ (B) and tris(bipyridine)ruthenium(ii) chloride (“Rubpy”) and Rubpy-MSN@TiO_2_ (C). Normalized fluorescence and fluorescence loss over irradiation time under UV-light is shown for sodium fluorescein and SG (D).

Fig. 4 compares fluorescence emission spectra of various dyes (C307, RhB and Rubpy, spectra of additional dyes are shown in Fig. S1†) and corresponding spectra of the dyes encapsulated in the MSN@TiO_2_ system. The spectral shifts seen upon encapsulation of the dye molecules can be explained by influence of the chemical surrounding,^39^*i.e.* replacing the water solvent with the silica-titania matrix or can be attributed to rigid framework of the carrier reducing rotational freedom of incorporated dyes.^67^ In both cases, the shifts indicate the successful encapsulation of the dyes.^68^ Corresponding to the peak shifts in fluorescence spectroscopy, shifts could also be observed in UV-Vis absorbance spectra (ESI Fig. S2†). Strongest peak shifts were found for R800-MSN@TiO_2_ and fluo-MSN@SiO_2_@TiO_2_ (Fig. S2A and B†). While the absorbance spectra of free rhodamine 800 dye shows both, monomer (at 689 nm) and dimer (at 629 nm), upon encapsulation a single peak is shifted to 618 nm, likely as a result of dimerization of the encapsulated dye molecules.^69^ For sodium fluorescein dye, absorbance peaks can be assigned to dianionic and monoanionic forms, while upon encapsulation only the dianionic absorbance peak with a minor shift is visible. This can be explained by the synthesis procedure: dianions preferentially form in basic environments while the occurrence of other forms increases with decreasing pH values.^70^ Based on the TGA results, an estimation of the loss of fluorescence intensity due to phenomena such as dye dimerization or absorption and scattering by the different particle layers can be performed. Comparison of the fluorescence intensity of a 10^−2^ mg mL^−1^ RhB-MSN@TiO_2_ and a free RhB solution with a concentration corresponding to the amount of encapsulated dye (1.9 × 10^−4^ mg mL^−1^) shows 10% fluorescence intensity for the encapsulated dye. Reduction in fluorescence signal originate from lower quantum yield of the encapsulated dye as well as absorption and scattering by the nanoparticle matrix. However, it should be noted that even with the reduction in fluorescence signal, the concentration of dye molecules in a confined space inside the particles results in a strong fluorescence signal. Achieving comparable signal intensities with free dye molecules in a large volume system such as a geological reservoir would require a very large amount of dye.

Sodium fluorescein is highly pH-sensitive and an additional silica shell between dye-MSN and titania layer was necessary to avoid contact of the acid used in titania layer synthesis and the encapsulated dye. This additional silica shell has proven to have further advantages as it helps reducing titania-mediated photocatalytically-induced degradation of the encapsulated dyes. The additional silica layer acts as a separation from the anatase crystallites, which are known to accelerate photodegradation under UV-light.^71,72^ Accelerated photodegradation was indeed observed for particles without the silica separation layer, such as SG-MSN@TiO_2_ (Fig. 4D). In contrast, in the case of fluo-MSN@SiO_2_@TiO_2_ and free sodium fluorescein, the encapsulated sodium fluorescein dye photodegradation is slowed.

### Stability, sorption and flow-through experiments

**Fig. 5:**
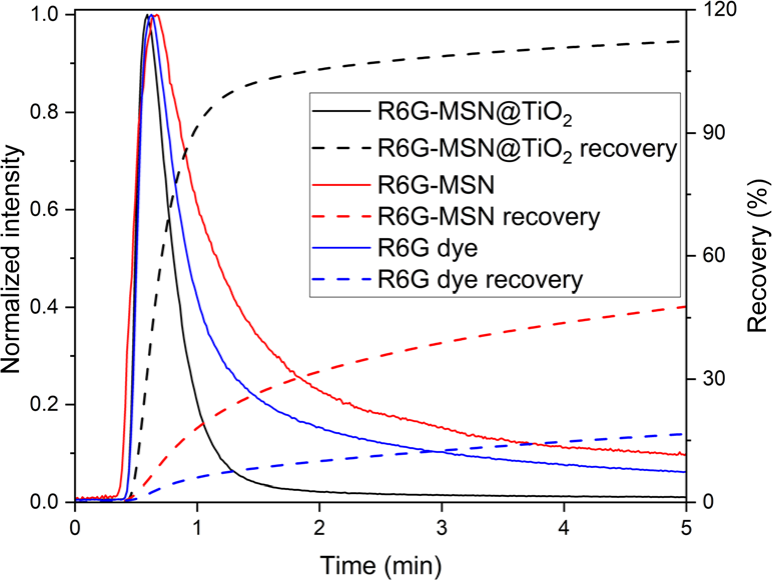
Flow-through quartz sand filled 25 cm column. Dye R6G is known to adsorb on quartz while R6G-MSN@TiO_2_ can penetrate through column. As reference for pore blocking ability of titania in R6G-MSN@TiO_2_, R6G-MSN are used without any coating.

Leaching tests were conducted to assess the possible payload leak due to imperfections in the titania encapsulation layer monitoring the fluorescence signal of the dye-loaded MSNs. Fluorescence intensity changes over 48 hours in Millipore water by average 4.77% ± 2.57% (*n* = 5), in 0.01 M NaCl by average 4.96% ± 2.95% (*n* = 10) and in 0.1 M NaCl by average 2.82% ± 1.71% (*n* = 8) ESI Table S1†) while monomeric silica concentration in solution (measured with photometry) remains in the solubility range of quartz (<10 mg L^−1^ at room temperature). These results underline the stability of the encapsulation method in order to prevent leakage of the dye and dissolution and disintegration of the silica carrier. In contrast, if the coating would not have been protective, stronger deviations in fluorescence intensity (as measured for free dyes in 0.01 M NaCl, Table S1 in ESI†) and increased monomeric silica concentrations due to carrier dissolution/disintegration could be expected.^16^ Flow-through experiments were conducted to further assess the encapsulation stability of the titania layer by comparing the transport properties of free dye, dye in MSN without encapsulation and encapsulated dye. Hereby, a cationic dye was chosen, as retention is expected to be high. Fig. 5 shows a comparison of the breakthrough curves of R6G dye, R6G-MSN and R6G-MSN@TiO_2_. R6G as a cationic dye is expected to adsorb on the negatively charged surface of the sand grains and indeed only 16.4% are retrieved within 5 minutes. Contrary, when dye is encapsulated inside MSN carrier coated with a protective titania layer the cationic dye is effectively shielded from its surrounding (as also corroborated by the ζ-potential measurements) and the adsorption depends on the outer titania layer. Consequently, sorption is dramatically reduced and the retrieval of R6G-MSN@TiO_2_ is nearly 7-fold that of the free R6G dye, with full recovery within 5 minutes. Furthermore, R6G-MSN@TiO_2_ show a shorter retention time and less pronounced tailing compared to dye breakthrough curve, which implies lesser dispersion and diffusion of the particles and proofs stable encapsulation of the dye. The stability of the encapsulation method is further highlighted by comparison with R6G-MSN without titania coating (Fig. 5): while the recovery is higher for dye-MSN than for free dye, the long tailing observed indicates flow of free dye molecules and is hence a strong sign of leakage.

### Comparison to other pore blockers

**Fig. 6:**
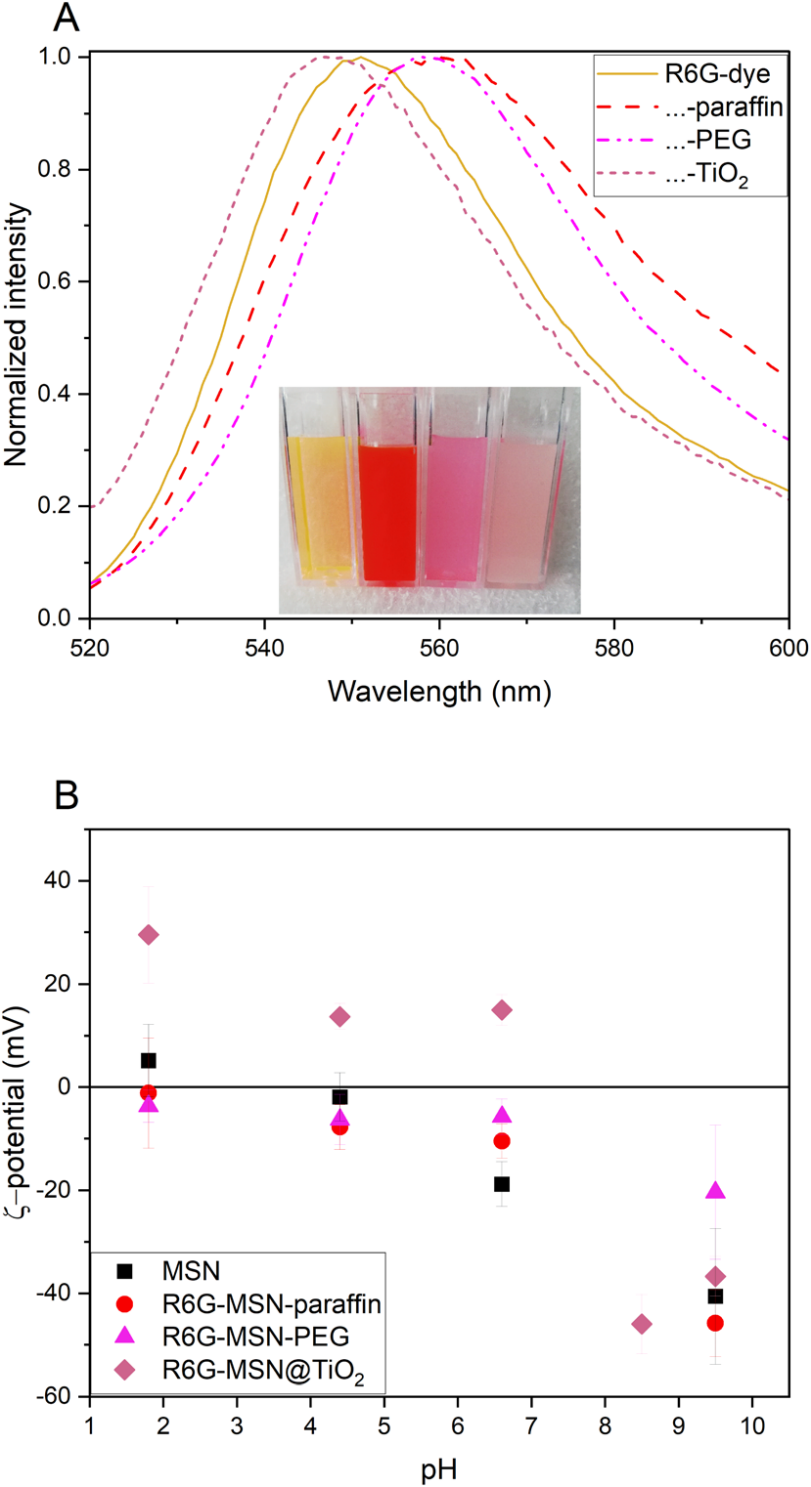
Fluorescence spectra (A) and ζ-potential (B) of hydrophilic, hydrophobic and metal oxide coating of mesoporous silica nanoparticles doped with rhodamine 6G dye. (A) Normalized fluorescence emission spectra for excitation wavelength at 480 nm. Bathochromic peak shift for PEG and paraffin coating 7 nm and 8 nm, respectively. Hypsochromic peak shift for titania coating 4 nm. Inset picture from left to right R6G-dye, R6G-MSN-C18-paraffin, R6G-MSN-SH-PEG and R6G-MSN@TiO_2_. (B) ζ-Potential of R6G-MSN@coating in comparison with carrier (MSN) over pH.

When a guest molecule is hosted inside a nanoparticle, the change in chemical environment and sterical effects lead to changes in guest molecule properties. In the case of loaded fluorescent molecules, this often manifests in shifts in the fluorescent emission wavelength. The addition of an encapsulation layer further changes the chemical environment and therefore causes an additional shift. The effect of titania coating is compared here to commonly used encapsulation strategies such as PEG or paraffin pore blocking. The thickness of the titania shell is with 7.1 nm comparable to the thicknesses of the organic modifications (5–15 nm).^33,73^ PEGylated MSN show hydrophilic surfaces while paraffin coated MSN are hydrophobic. Fluorescence spectra R6G-dye, R6G-MSN@TiO_2_, R6G-MSN-SH-PEG, and R6G-MSN-C18-paraffin is shown in Fig. 6. It can be seen that PEG and paraffin induce a bathochromic peak shift while titania induces a hypsochromic one. The shift can also be seen by the naked eye, as is shown in the inset picture in Fig. 6A. In terms of ζ-potential, the titania coating leads to positive ζ-potentials at pH 6.6 in 10 mM KCl while PEG- and paraffin-coated particles still show negative ζ-potentials under same conditions. Having positive ζ-potential bearing particles can favor for example their cellular uptake.^7^

## Conclusion

The here presented titania-mediated encapsulation method for fluorophores inside silica nanocarriers enables synthesis of highly stable and robust fluorescent nanoparticles. The synthesis chosen for this purpose utilized a reverse (water-in-oil) microemulsion environment, adapted for the size of the carriers, which facilitated selective titania growth in separate droplets while avoiding merging of particles. The synthesis has proven to be highly reproducible, which is another known advantage of using microemulsions,^74^ and was found to be applicable for a wide range of payloads. Capping the 140 nm-sized MSN, the synthesized titania layer has a thickness of about 7.1 ± 4.3 nm, which XRD shows consist of a mixture of amorphous titania and anatase. Bonding of the titania layer to the silica surface is confirmed by formation of Ti–O–Si bonds as can be learned from FT IR spectroscopy. Leaching tests and flow-through experiments further show that the payload is effectively encapsulated. The band gap of anatase with 3.2 eV prohibits usage of UV-fluorophore, but does not impact visible light fluorophores. Moreover, the ability of titania to absorb and scatter UV-light can be a challenge if encapsulated fluorophores are prone to photodegradation (such as sodium fluorescein). A prevention strategy could be the addition of a silica shell between dye-MSN and titania layer that reduces photodegradation under UV-light, which can be of particular interest in hydrology applications.

It should be noted that while the titania layer provides a robust coating to endure harsh ambient conditions, it comes with the drawback that its robustness does not enable a stimuli-responsive degradation, rendering the system impractical for release applications.

Just like as in the case of silica, titania also presents a high density of hydroxyl groups on its surface, making it facile for a wide array of surface modification of particles.^75^ Additionally, recent studies suggest that titania coating improve the biocompatibility of nanoparticle carriers,^29^*i.e.* using titania as pore blocker could be useful in biological and biomedical applications. This makes the here presented particles to be of particular interest as bio- and geoimaging tracers as they can be applied regardless of ambient conditions due to the stable and robust nature of the titania coating layer.

## Conflicts of interest

There are no conflicts to declare.

## Supplementary Material

NA-006-D4NA00242C-s001
