# Supplementary material for: Titania-mediated stabilization of fluorescent dye encapsulation in mesoporous silica nanoparticles
Source: Nanoscale Adv. 2024 May 29;6(13):3450–61. doi: 10.1039/d4na00242c (PMC11197426; doi:10.1039/d4na00242c)
Supplement: NA-006-D4NA00242C-s001 [file NA-006-D4NA00242C-s001.pdf]

Electronic Supplementary Material (ESI) for Nanoscale Advances.
This journal is © The Royal Society of Chemistry 2024

Supplementary Material:

Titania-Mediated Stabilization of Fluorescent Dye Encapsulation in Mesoporous Silica Nanoparticles

Laura Spitzmüller, Jonathan Berson, Fabian Nitschke, Thomas Kohl, Thomas Schimmel

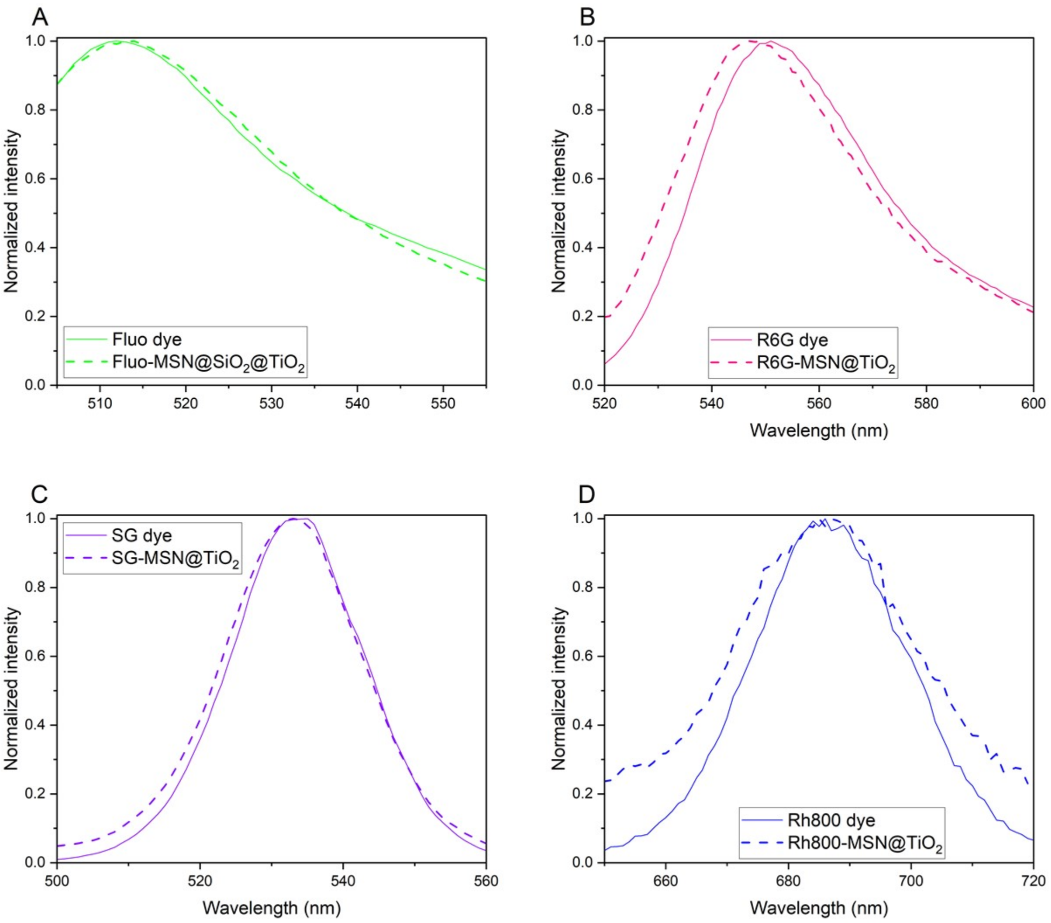

Figure S 1: Fluorescence emission spectra for sodium fluorescein/uranine/Fluo and Fluo-MSN@SiO_2@TiO_2 (A), synchroscan spectrum (lambda 20) for rhodamine 6G and R6G-MSN@TIO_2 (B), synchroscan spectrum (lambda 20) sulforhodamine G and SG-MSN@TiO_2 (C) and synchroscan spectrum (lambda 23) for rhodamine 800 and Rh800-MSN@TiO_2 (D).

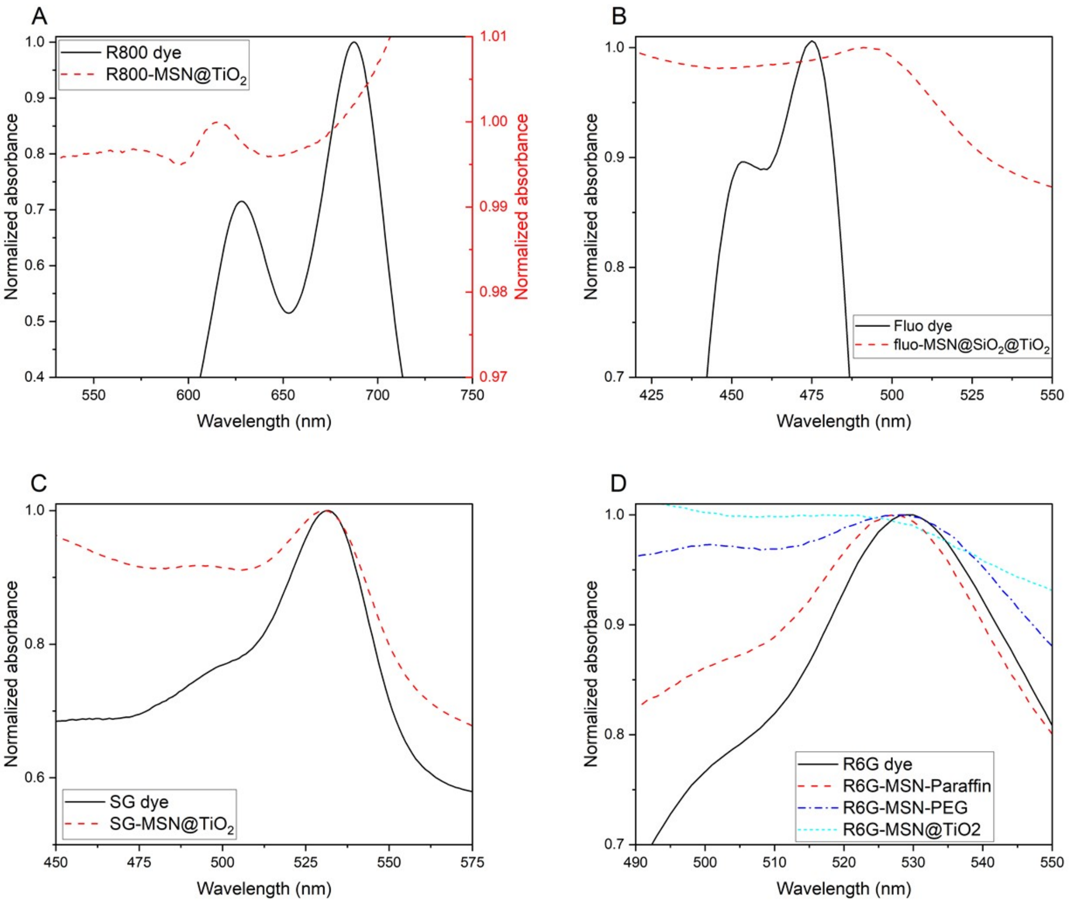

Figure S 2: Absorbance spectra: R800: Peak dye 629 nm (dimer) and 689 nm (monomer), R800-MSN@TiO_2 617 nm (dimer). Fluorescein peak from mono-and dianionic forms, peak shift fluo-MSN@SiO_2@TiO_2 corresponds to dianionic form. SG and SG-MSN@TiO_2, R6G and R6G-MSN@TiO_2 show only minor peak shifts.

Table S 1: Stability of fluorescence intensity of dye-MSN@TiO_2 and dye in different solutions over time.

Sample	Solution	Fluorescence intensity at time 0	Fluorescence intensity after 48 hours	Difference (%)
Rubpy-MSN@TiO_2	Millipore water	217.97	209.86	3.7
Rh800-MSN@TiO_2	Millipore water	458.74	483.85	5.5
R6G-MSN@TiO_2	Millipore water	130.41	135.93	4.2
RhB-MSN@TiO_2	Millipore water	287.67	284.02	1.3
SG-MSN@TiO_2	Millipore water	126.94	139.71	9.1
R6G-MSN@TiO_2	0.01M NaCl	243.89	238.86	2.1
R6G-MSN@TiO_2	0.01M NaCl	274.07	281.09	2.6
R6G-MSN@TiO_2	0.01M NaCl	271.22	284.77	5.0
RhB-MSN@TiO_2	0.01M NaCl	514.55	558.97	8.6
RhB-MSN@TiO_2	0.01M NaCl	521.07	522.12	0.2
RhB-MSN@TiO_2	0.01M NaCl	272.58	256.37	5.9
RhB-MSN@TiO_2	0.01M NaCl	657.08	724.38	10.2
SG-MSN@TiO_2	0.01M NaCl	639.12	667.36	4.4

SG-MSN@TiO_2	0.01M NaCl	420.96	434.96	3.3
SG-MSN@TiO_2	0.01M NaCl	533.25	571.53	7.2
R6G-MSN@TiO_2	0.1M NaCl	276.33	283.04	2.4
R6G-MSN@TiO_2	0.1M NaCl	313.24	319.64	2.0
R6G-MSN@TiO_2	0.1M NaCl	311.01	314.86	1.2
RhB-MSN@TiO_2	0.1M NaCl	551.30	573.99	5.4
RhB-MSN@TiO_2	0.1M NaCl	488.26	508.33	5.5
SG-MSN@TiO_2	0.1M NaCl	714.82	709.33	0.8
SG-MSN@TiO_2	0.1M NaCl	742.28	769.30	3.6
SG-MSN@TiO_2	0.1M NaCl	723.21	734.68	1.5
R6G dye	0.01M NaCl	439.12	272.52	37.9
R6G dye	0.01M NaCl	451.18	271.23	39.9
RhB dye	0.01M NaCl	430.30	357.39	16.9
RhB dye	0.01M NaCl	396.75	246.17	38.0
RhB dye	0.01M NaCl	409.32	250.28	38.9
SG dye	0.01M NaCl	448.98	467.17	4.1
SG dye	0.01M NaCl	499.32	522.26	4.6
SG dye	0.01M NaCl	434.10	463.33	6.7
R6G dye	0.1M NaCl	612.83	646.30	5.5
R6G dye	0.1M NaCl	773.09	680.35	12.0
RhB dye	0.1M NaCl	691.79	694.53	0.4
RhB dye	0.1M NaCl	571.79	576.54	0.9
SG dye	0.1M NaCl	203.70	208.70	2.5
SG dye	0.1M NaCl	211.22	213.33	1.0

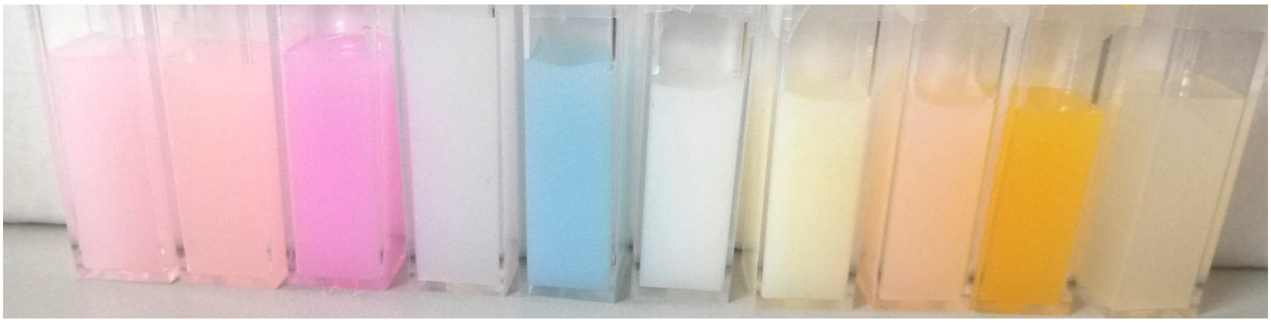

Figure S 3: Photograph of dye-MSN@TiO_2. From left to right: R6G-MSN@TiO_2, SG-MSN@TiO_2,RhB-MSN@TiO_2, C307-MSN@TiO_2, NBA-MSN@TiO_2, 1,3,6-naphthalene sulfonate NTS-MSN@TiO_2 (UV-Tracer, therefore no color visible and no fluorescence detected), Fluo-MSN@SiO_2@TiO_2, Rubpy-MSN@TiO_2, Rubpy@SiNPs@SiO_2, Fluo-MSN@TiO_2.

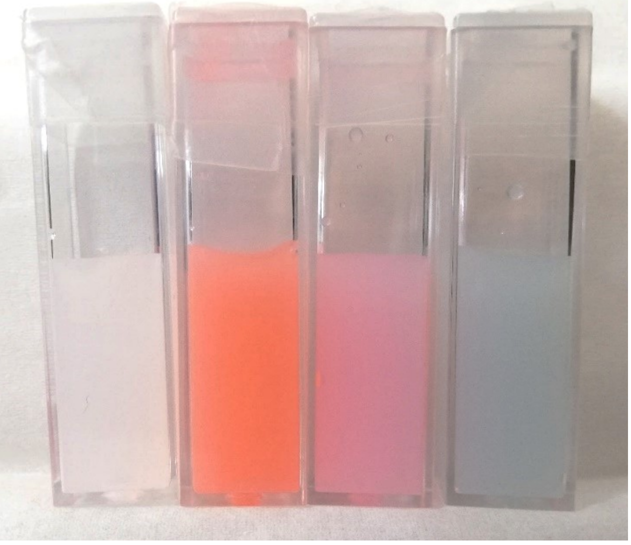

Figure S 4: Photograph of pure titania particles, SG-MSN@TiO_2, R6G-MSN@TiO_2, R800-MSN@TiO_2 (from left to right).
